# Short PolyA RNA Homopolymers Undergo Mg^2+^-Mediated Kinetically Arrested Condensation

**DOI:** 10.1021/acs.jpcb.2c05935

**Published:** 2022-11-15

**Authors:** Jenna K. A. Tom, Paulo L. Onuchic, Ashok A. Deniz

**Affiliations:** Department of Integrative Structural and Computational Biology, The Scripps Research Institute, 10550 North Torrey Pines Road, La Jolla, California92037, United States

## Abstract

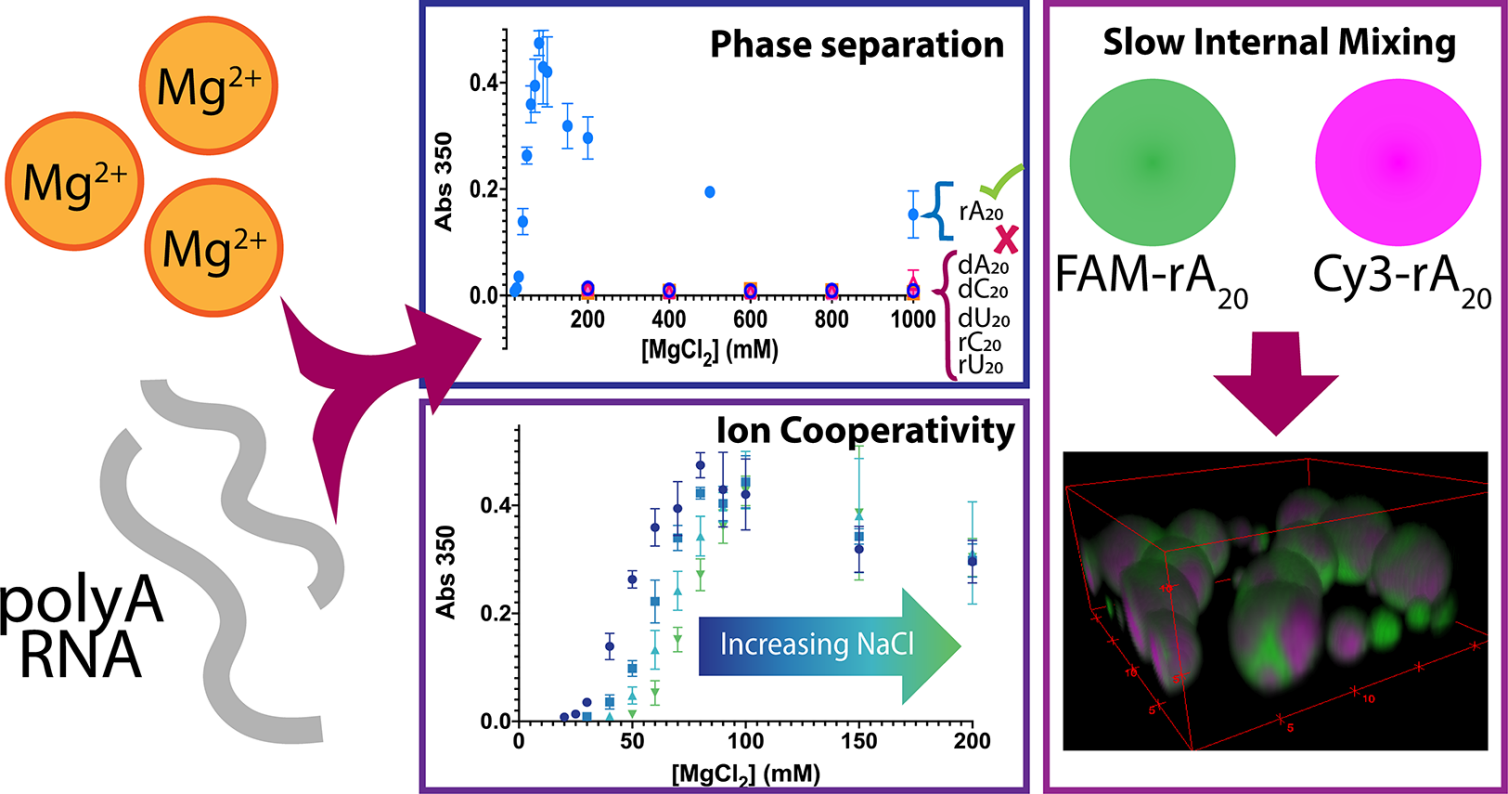

RNA–RNA interactions have increasingly been recognized for their potential to shape the mesoscale properties of biomolecular condensates, influencing morphology, organization, and material state through networking interactions. While most studies have focused on networking via Watson–Crick base pairing interactions, previous work has suggested a potential for noncanonical RNA–RNA interactions to also give rise to condensation and alter overall material state. Here, we test the phase separation of short polyA RNA (polyrA) homopolymers. We discover and characterize the potential for short polyrA sequences to form RNA condensates at lower Mg^2+^ concentrations than previously observed, which appear as internally arrested droplets with slow polyrA diffusion despite continued fusion. Our work also reveals a negative cooperativity effect between the effects of Mg^2+^ and Na^+^ on polyrA condensation. Finally, we observe that polyrA sequences can act as promoters of phase separation in mixed sequences. These results demonstrate the potential for noncanonical interactions to act as networking stickers, leading to specific condensation properties inherent to polyrA composition and structure, with implications for the fundamental physical chemistry of the system and function of polyA RNA in biology.

## Introduction

The formation of bimolecular condensates (BMCs) through phase separation and percolation-related processes has been linked to many regulatory processes in cells, including cellular responses to stress, development, and disease.^1−6^ Characterized as non-stoichiometric assemblies of nucleic acids, proteins, and other macromolecules,^5,7^ such condensates present an alternative mechanism beyond membrane-enclosed organelles for potential dynamic compartmentalization in cells.

RNA and RNA-binding proteins are among the most abundant molecules in biomolecular condensates. Several cellular ribonucleoprotein (RNP) granules are enriched in these molecules, making them targets of interest in understanding how and why condensates form. There is much evidence that multivalent interactions between RNA and proteins can lead to phase separation. These interactions can include specific interactions with RNA-binding proteins or nonspecific interactions with charged intrinsically disordered proteins (IDPs). While many phase separation studies have focused on IDPs and the necessity of key proteins in RNP granules, the significance of RNA in BMCs is increasingly being recognized.^8−11^ Recent work has shown that RNA can not only drive phase separation but also play a role in condensate viscoelasticity and gelation,^12−16^ morphology,^14,16−19^ organization,^20−22^ and client partitioning^12,23,24^ through a variety of interaction-based mechanisms. These include the inhibition^25^ and induction^26^ of conformational changes in protein binding partners, the formation of Watson–Crick interactions in repeat sequences,^15^ electrostatic interactions with polycation partners,^12,13,16,27^ and potential structure-induced effects.^12,18,28^

Among these studies is a growing body of evidence that RNA–RNA interactions can drive condensate formation and influence the mesoscale properties of complex systems.^8,15,16,18,29−31^ While several studies have focused on the role of Watson–Crick-based interactions,^15,18,29^*in vitro* studies have also shown that noncanonical RNA interactions have the capacity to drive phase separation and tune the material and compositional properties of RNA condensates.^16,17,23,29−31^ Previous work has demostrated that even RNA homopolymers can phase separate in the presence of high concentrations of salt, likely mediated by noncanonical RNA–RNA interactions.^16,17,23,30^ In such homopolymer-based systems, the identity of the homopolymers has a dramatic influence on the phase behavior. In the presence of PEG and high concentrations of NaCl, long RNA polymers of polyrU, polyrC, and polyrA condense to form droplets with distinct properties, while polyrG forms aggregates, presumably due to its tendency to form stable G-quadruplex structures in the presence of monovalent salts.^30^ Other studies have also shown that polyrU can phase separate in the absence of PEG when mixed with sufficient concentrations of divalent cations^17^ and that differences in scaffold identity influence partitioning and organization.^23^ These findings and others in the field have suggested that noncanonical RNA–RNA interactions and the chemical properties of constituent polymers influence the phase behavior and physical properties of RNA condensates.

Of these homopolymers, polyrA is especially interesting from both biological and chemical perspectives. Physiologically, long tracts of polyadenylated tails are highly conserved, making it among the most universally conserved homopolymer present in cells. The length and presence of these sequences have been functionally associated with mRNA half-life, degradation machinery, and transcriptional processes.^32,33^ From a chemical perspective, polyrA, despite its simplicity of sequence, has a complex array of structural forms. Due to its potential for self-interaction, polyrA has the potential to transition between random coils, single-stranded helices, and double-stranded structures depending on environmental conditions.^34−40^ Furthermore, recent studies indicate that polyrA interacts with Mg^2+^ in a manner distinct from polyrU and polydA with respect to both hydration and binding preference compared to Na^+^.^41,42^

In this paper, we explore the ways in which short polyrA sequences can also exhibit interesting phase behavior. Work as far back as 50 years ago showed that polyrA sequences can form helical structures and precipitate in the presence of monovalent salt.^34,35^ Recent work has shown that these precipitates can be liquid-like condensates with long polyrA sequences in the presence of crowders.^23,30^ Other studies have also highlighted the unique role of Mg^2+^ in interacting with polyrA to form aggregates with both long polyrA^41^ and short polyrA.^42^ Despite the potential for interesting features in polyrA phase separation, Mg^2+^-mediated polyrA droplet formation has not yet been characterized for short polyrA sequences. This presents an opportunity to explore the limits of RNA phase separation and to further probe the implications of polyrA–Mg^2+^ interactions in the context of phase separation.

Here we use 20-mer polyrA oligomers (rA_20_) to characterize the phase separation properties of shorter homopolymeric polyrA. We found that rA_20_ phase separates into condensates at substantially lower Mg^2+^ concentrations than previously observed in longer polyrU sequences. Our results revealed that these condensates display properties consistent with kinetically arrested states with slow internal rearrangement. We also discovered an unexpected cooperativity effect on rA_20_ condensation between Mg^2+^ and Na^+^. Additionally, given the importance of polyrA tails in biology and the unique phase properties we observed of rA_20_, we hypothesized and tested its ability to act as a phase separation promoter when tagged onto a generic mixed RNA 20-mer construct (rN_20_), comparing 40-mers with tails of rA_20_ to those with tails of rU_20_ and the mixed sequence rN_20_.

## Materials and Methods

### Sample Preparation

All oligomers (rC_20_, rU_20_, rA_20_, dC_20_, dT_20_, dA_20_, rA_15_, rA_10_, rA_25_, rA_30_, FAM-A_20_, Cy3-rA_20_, FAM-rN_20_ + rA_20_, and rN_20_ + rA_20_-Cy5) were ordered from Sigma-Aldrich (Texas, USA) with HPLC purification. These were resolubilized in diethyl pyrocarbonate (DEPC) treated water (Santa Cruz Biotech) and diluted into buffer (Tris-HCl, pH 7.5). Solids of MgCl_2_ and NaCl purchased from Sigma-Aldrich (Texas, USA) as well as polyethylene glycol (PEG, MW 8000, Biotech) were dissolved in DEPC treated water.

### Condensate Formation

To solutions of nucleic acids in buffer (Tris-HCl, pH 7.5), varying concentrations of 2 × MgCl_2_ solution were added to reach the final concentrations of the nucleic acids in 50 mM Tris-HCl, pH 7.5. The final concentration of rA_20_ was 0.5 mg/mL, and other 20-mer nucleic acids were mixed to reach the molar equivalent (∼77 μM) unless otherwise specified. Concentrations of the master mix of nucleic acid and buffer before condensation were checked by a 5- or 10-fold dilution using A260. The 40-mer mimic constructs were made so that the total amount of bases in solution was comparable to the 20-mer experiments(∼38 μM). For experiments with PEG or monovalent salt, a 4X nucleic acid master mix was made and aliquoted. The resultant mixtures were then diluted 2-fold with the respective additive and mixed. All nucleic acid master mixes were allowed to equilibrate to room temperature before a final step of MgCl_2_ addition, which brought concentrations to the final values recorded.

### Absorbance and Turbidity

UV–vis absorption spectra were recorded with a Nanodrop 2000c. Absorbance at 350 nm was used to report on the formation of aggregates. Data shown represents triplicate runs where MgCl_2_ and nucleic acid master mixes were remade, with error bars depicting the standard deviation of values between the runs.

### Droplet Microscopy

All differential interference contrast (DIC) and fluorescence microscopy images were recorded on a Zeiss LSM 780 laser scanning confocal microscope. Samples were imaged at room temperature using a 100× oil immersion objective (Plan-Apochromat 100×/1.40 Oil DIC M27) with a scan speed of 314 ms/pixel and a pixel size of between 0.17 and 0.08 μm. For DIC images, 40% of 488 nm laser power was applied, and averaging was set to 4. In FRAP and fusion-based studies, averaging was turned off and lower laser power settings were applied to mitigate the effect of photobleaching over time. For 6FAM-rA_20_ labeled condensates, 0.3% of 488 nm laser power was applied, while for Cy3-rA_20_ labeled condensates, laser power was 0.8% at 514 nm for standard readings. In readings where time series were applied over a period of time greater than 10 min, reflection autofocus was turned on and was applied every 5 min.

Fluorescently labeled oligomers and dyes were added prior to condensate formation such that their final concentrations were 1 μM. After the addition of MgCl_2_, samples were mixed and subsequently transferred to Lab-Tek chambered no. 1.5 borosilicate cover glass which had been treated with 10% TWEEN-20 for 35 min, rinsed with DEPC treated water, and dried overnight prior to storage and use. Droplets were formed separately within a minute of each other and then sequentially added to the coverslip after about 10–30 min of incubation on the cover glass after droplet formation as specified below.

All images and videos were analyzed with Fiji software.

### FRAP

For fluorescence recovery after photobleaching (FRAP) experiments, samples were prepared in the same manner as described above, with the 6FAM-rA_20_ oligomer used as a marker. Droplets were partially bleached (region of interest (ROI) area ∼ 0.35 μm^2^ in droplets with radii approximately 5-fold the bleached radii). Experimental droplets were bleached using four to six iterative pulses of 100% 488 and 405 laser power, after which the bleached area was below 50% of its initial signal (typically to approximately 40%). Images were corrected for *xy* drift by use of the Correct 3D drift option on the Fiji Registration plug-in.^43^ For a few images with more *xy* drift, the StackReg plug-in was first applied with the Rigid Body correction^44^ and Correct 3D drift was applied after.

Fluorescence recovery of the bleached area was referenced to an area inside an unbleached droplet of a comparable size to account for photobleaching and to calculate a normalized intensity:
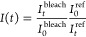
For the rN_20_ + rA_20_ constructs, normalized curves were plotted and fit to an exponential curve using GraphPad Prism, *y* = *y*_0_ + *A*(1 – e^*t*/τ^), from which the immobile fraction was calculated with the equation , where *A* corresponds to the empirical fit parameter. Multiple droplets (*n* = 3) were used for each condition. For a detailed description of FRAP conditions, please see the Supporting Information.

### Coalescence and Mixing

To visualize droplet fusion and mixing, two sets of samples were separately prepared in the same manner as described above, with the use of either the 6FAM-rA_20_ or Cy3-rA_20_ oligomer as a marker. Droplets were formed with either the Cy3 or FAM labeled oligomers nearly simultaneously. The Cy3-rA_20_ was then transferred to the cover glass and used to focus the confocal microscope on the *z*-dimension of interest. An image was collected each minute starting from 15 min after formation and incubation of Cy3-rA_20_ for a total of 60 min. After 5 min, FAM-rA_20_ was then added to the top of the mixture. Fusion of these droplets just above the cover glass was observed. Three-dimensional images were rendered with the 3D Viewer Plugin on Fiji/ImageJ.

## Results and Discussion

### Short PolyrA Forms Cation-Induced Homotypic RNA Condensates

While previous studies have shown the ability of long RNA homopolymers to phase separate in the presence of high concentrations of cations and, in some cases, crowder,^17,23,30^ the extent to which these homopolymers could phase separate given shorter, fixed lengths was yet unknown. To test the potential for short, nucleic acid oligomer phase separation, we compared the phase separation of 20-mer RNA constructs (rA_20_, rC_20_, and rU_20_) and corresponding DNA counterparts (dA_20_, dC_20_, and dT_20_) in the presence of Mg^2+^ with increasing divalent cation concentrations up to 1 M Mg^2+^. Using DNA and RNA forms of equal length allowed for comparison across different polymers given variations in both nucleobase and backbone sugar structures. Although they would have been a useful data point, polyG oligomers were not tested here given difficulties in acquiring oligomers on this length scale.

To measure condensate formation, we used a combination of absorption spectroscopy and confocal microscopy. Changes in absorbance at 350 nm, a region where no native nucleic acid or Mg^2+^ absorption was observed, were used as a proxy for condensate formation as previously reported.^14,17^ Droplet formation and morphology were confirmed with DIC imaging for select points in the data set.

**Figure 1:**
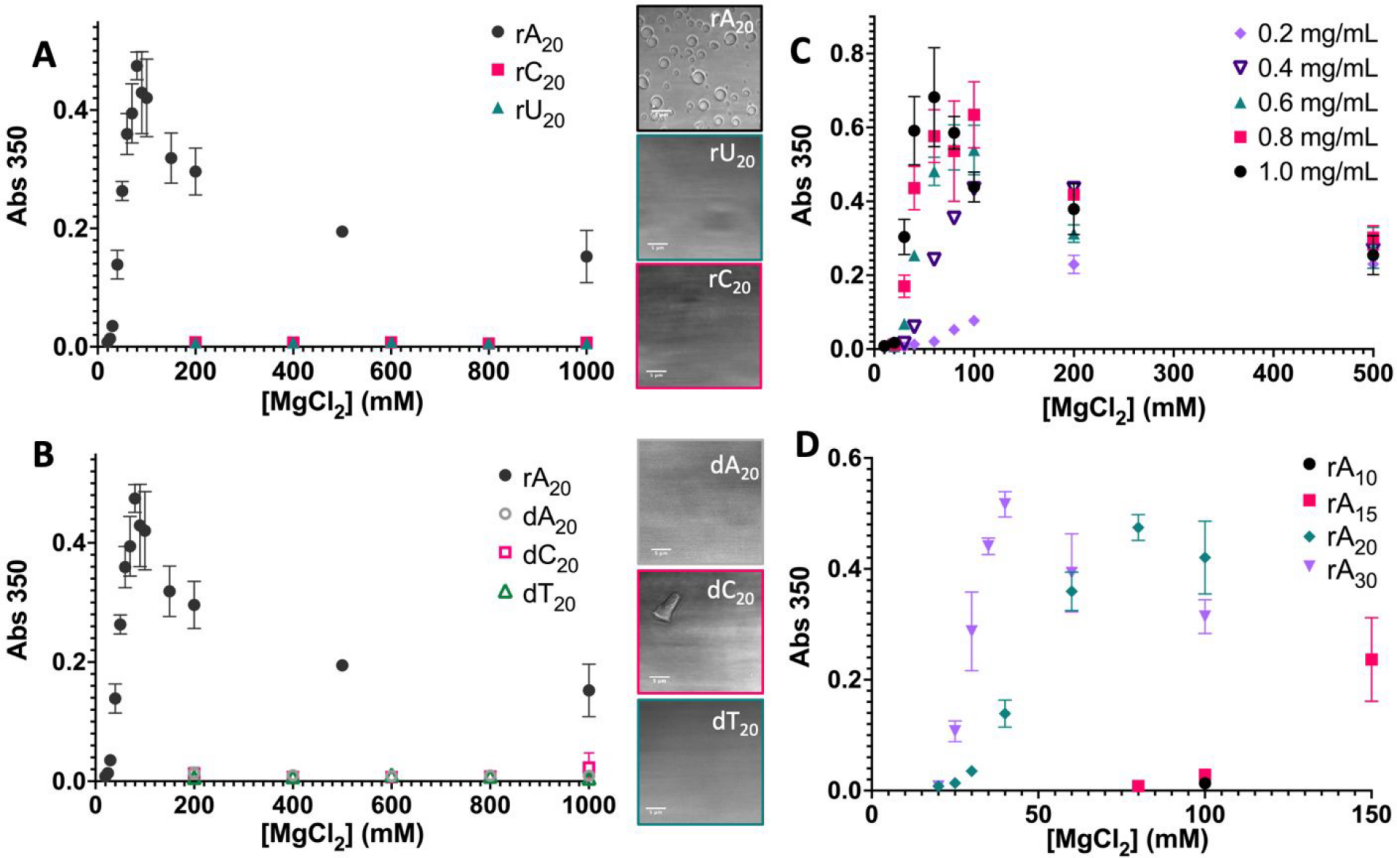
PolyrA_20_ undergoes Mg^2+^-mediated phase separation. (A) PolyrA_20_ (77 μM, 50 mM Tris, pH 7.5) condenses into spherical droplets in the presence of Mg^2+^, while polyrU_20_ and polyrC_20_ do not (scale bars = 5 μm, [Mg^2+^] = 200 mM). (B) Comparatively, no DNA oligomers form spherical condensates under the same conditions. Threshold Mg^2+^ needed to undergo phase separation (C) increases with rA_20_ concentration and (D) decreases with rA length for a constant amount of polyrA_*n*_ (0.5 mg/mL).

Our results indicated that short polyrA_20_ begins phase separating at 30 mM Mg^2+^, which we throughout this paper refer to using the notation, “≥30 mM Mg^2+^”, marking the lowest Mg^2+^ concentration tested where absorbance at 350 nm increases above background levels (Figure 1A). This is nearly an order of magnitude lower Mg^2+^ concentration than the previously published 250 mM Mg^2+^ for long polyrU (800–1000 kDa) in the absence of a crowder.^17^ It is also significantly lower than the concentration of monovalent salt noted in the phase separation observed with long polyrA in the presence of 750 mM NaCl with a crowder.^23,30^ Interestingly, the transition appears to show nonmonotonic behavior, where the curve increases from about 30 mM Mg^2+^, passes through a maximum around 80 mM, and then subsequently decreases. Previous examples of nonmonotonic, reentrant behavior in multicomponent systems have been observed for example in charge-based systems,^14,45^ where a balanced ratio of charged species tends to favor separation and strongly unbalanced systems do not due to charge repulsion and screening of networking interactions. In this way, the presence of reentrant behavior could be indicative of multiple competing forces involved in the phase separation of polyrA and Mg^2+^ which include both charge-based networking interactions and the more hydrophobic rA–rA interactions that are not screened by salt. Just as in the reentrant systems of charged polymer species, we hypothesize that it is possible for rA–Mg^2+^ systems to have an optimal balance of these interactions based on the concentrations of rA and Mg^2+^. However, it is also possible that rather than the quantity of material phase separated, the size and shape of the droplet profile are driving the nonmonotonic behavior. Either of these possibilities is interesting and could warrant future exploration.

Additionally, none of the other short RNA oligomers that were tested showed significant changes in absorbance (Figure 1A), nor did any of the other DNA oligomers, although dC_20_ did form sparsely populated irregular aggregates (Figure 1B). In this way, polyrA appears to have some intrinsic characteristic enabling phase separation in the presence of Mg^2+^ at relatively low concentrations given the short length regime that neither the pyrimidine nor its DNA counterpart possesses. The observed droplet formation is thus neither fully a property of RNA nor a general property of its specific nucleobase, but rather a combination of both.

Given that rA_20_ is already a short polymer relative to most previous homotypic examples of condensate formation, we were interested in probing the limits of its phase separation given further changes in concentration and length. For homopolymer systems, it is expected that increases in length and concentration increase phase separation propensity.^7^ Such an increase in phase separation propensity would correlate with a decrease in threshold divalent cation concentration needed to induce phase separation observed by a shifting of the absorbance curve to the left.

Predictably, we observed a shift in the observed phase boundary toward lower Mg^2+^ concentration with increasing rA_20_ concentration. The sample at 0.2 mg/mL phase separated at the highest concentration of divalent cation (≥60 mM Mg^2+^), while all samples at 1.0, 0.8, and 0.6 mg/mL phase separated at ≥30 mM Mg^2+^, with the intensities of absorbance at 350 nm increasing with concentration and likely suggesting an earlier onset of condensation (Figure 1C). Thus, like RNA in other phase separating systems, RNA concentration is a tuner of this system. It is also worth noting that, even at the lowest concentration tested, 0.2 mg/mL (∼31 μM), phase separation at 60 mM Mg^2+^ is still comparatively lower than the previously reported homotypic RNA phase separation in the presence of cations despite the comparatively reduced concentration. In the presence of 5 and 10% PEG-8000, the boundary can shift such that phase separation is observed at ∼20 mM Mg^2+^ at a concentration of 0.5 mg/mL instead of 30 mM Mg^2+^ (Figure S1), further reducing Mg^2+^ thresholds.

In addition to investigating the effects of RNA concentration on the phase separation of the system, we were also interested in determining the effects of length on the phase boundary and observing whether there was a lower bound to phase separation given a lower valency. Previous fractionation of long polyrA showed a positive relationship between polymer length and phase separation propensity given phase separation with NaCl and temperature.^34^ We expected a similar relationship to occur with MgCl_2_ addition with the possibility for such a reduced length to not phase separate under the conditions established. To test this, we examined the phase separation potential of rA_10_, rA_15_, rA_20_, and rA_30_ where the final concentration of each species was adjusted to approximately 0.5 mg/mL, the equivalent concentration used for rA_20_ in the RNA/DNA base comparisons. Consistent with previous studies which demonstrated the importance of multivalency in phase separation,^46,47^ our data showed that short polyrA condensation is also a length dependent process, with increasing length favoring phase separation and shifting the phase boundary toward lower Mg^2+^ concentrations. The shortest length polyrA to phase separate under these conditions was rA_15_, which phase separated around 100 mM Mg^2+^, followed by rA_20_ (≥30 mM Mg^2+^) and rA_30_ (≥25 mM Mg^2+^) (Figure 1D) with rA_10_ not showing significant changes in A350 under 500 mM Mg^2+^ (Figure S2). This is consistent with the idea that, below a critical length, RNA no longer forms liquid droplets^29^ under these conditions and shows that even as few as 15 nucleotides of polyrA are sufficient to observe phase separation within the polyrA/Mg^2+^ concentration regime tested.

These observations of length, concentration, and crowding are consistent with the current understanding of the importance of valency, concentration, and crowding in tuning phase boundaries and condensation. The fact that Mg^2+^-mediated polyrA condensation does appear to follow these expectations makes it more striking that rA_20_ comparatively phase separates at such low concentrations of Mg^2+^ in the absence of crowders and at lower lengths and concentrations compared to previously demonstrated long homopolymer work.^16,17,23,30^ That this is unique to polyrA of the nucleic acids tested is also interesting in part because of the aforementioned differences in how it interacts with Mg^2+^ when compared to both polyrU and its DNA analogue^41,42^ as well as its potential for self-interaction.^34−40^ Networking and phase transitions rely upon favorable interactions with other macromolecules.^5,7^ In this sense, the same π–π and cation−π interactions that enable the formation and destabilization of secondary structural elements with itself given changes in environment both with cations and with pH^34−39,48,49^ could be related to polyrA’s ability to phase separate under relatively low Mg^2+^.

### PolyrA Condensates Form Spherical Droplets but Show Limited Mixing

**Figure 2:**
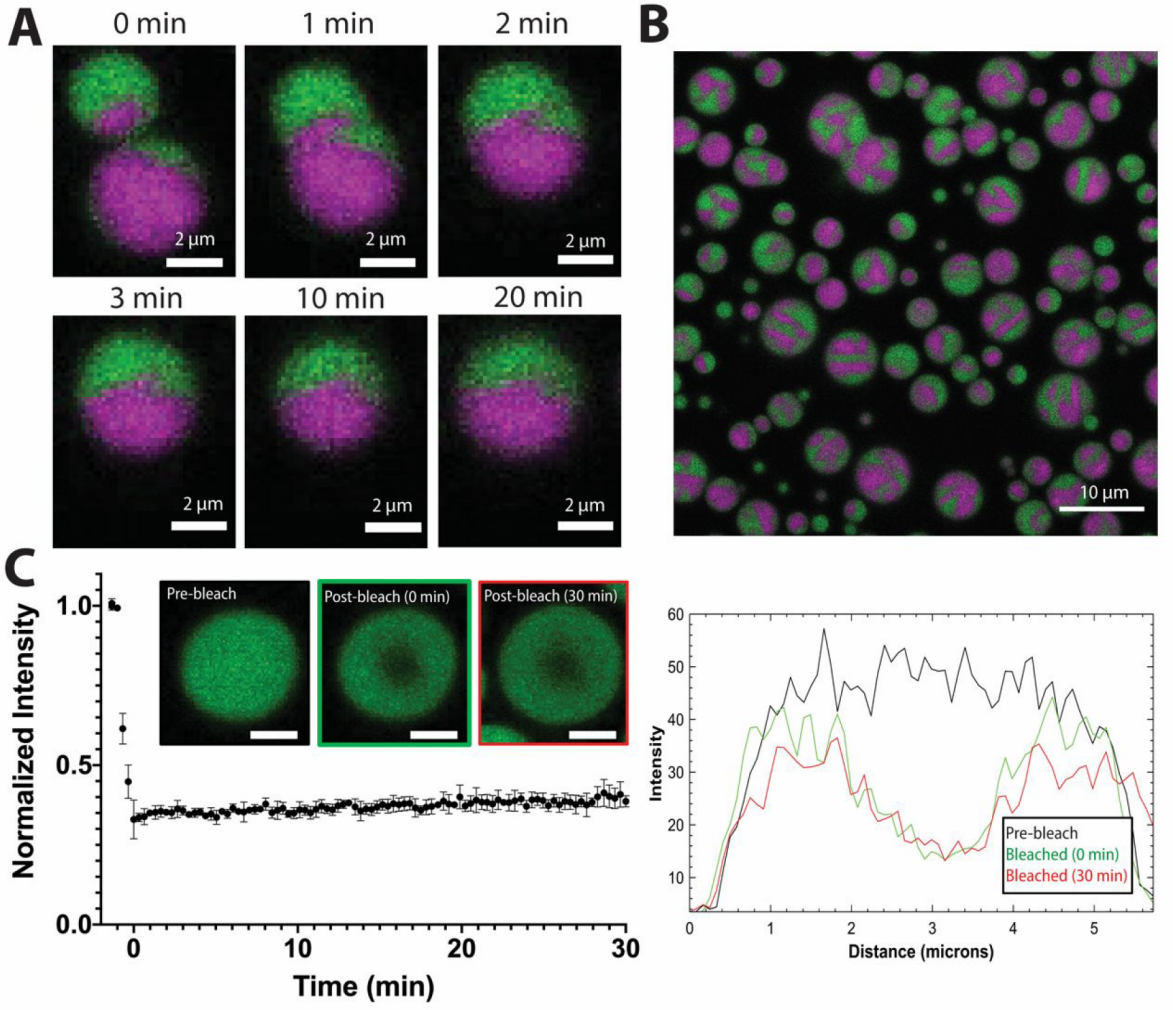
PolyrA_20_ shows slow internal dynamics and mixing (0.5 mg/mL polyrA_20_, 80 mM Mg^2+^, 50 mM Tris-HCl, pH 7.5). Droplet formation was induced with Mg^2+^ addition separately such that droplets initially were labeled with only one dye labeled species (FAM-rA_20_ or Cy3-rA_20_). FAM-labeled droplets were added first to the cover glass. Cy3-labeled droplets were added 15 min after droplet formation, . (A) Fusion of two rA_20_ droplets from the differentially labeled FAM-rA_20_ (green) or Cy3-A_20_ (magenta) populations over the course of 20 min (scale bar = 1.5 μm). The time *t* = 0 specifies the time just before this fusion event starts. This correlates with a time approximately 7 min after addition of Cy3-A_20_ labeled droplets and 22 min after droplet formation with Mg^2+^ addition. (B) After 70 min post addition of Cy3-A_20_ droplets, residual fusion events can be observed by the incomplete mixing of fused droplets. The image is taken at approximately 3 μm above the coverslip from a *z*-stacked image (Figure S4). (C) Average recovery of normalized FAM-rA_20_ intensity; the bleached ROI in a FAM-rA_20_ labeled condensate is shown. The inset depicts a representative droplet at three different time points (prebleached, immediately after bleaching, and at 30 min after bleaching (scale bar = 2 μm)). A line scan through the center of the droplet is shown to the right highlighting the bleach profile.

Like other liquid-like condensates, polyrA droplets merge upon contact and form spherical droplets (Figure 2A). In previous studies, this spherical formation has been considered a qualitative mark of liquid-like properties.^50^ However, unlike typical liquid-like condensates, these rA_20_ droplets seem to exhibit very slow mixing and internal exchange while still fusing into spherical droplets. Here, we use “fusion” to refer to the process of droplets combining to form a single droplet separate from the process of internal content mixing.

To visualize the fusion process, we monitored rA_20_ droplets separately labeled with two different dyes, 6FAM-rA_20_ and Cy3-rA_20_. The fluorescent markers were added to the sample prior to droplet formation to a final concentration of about 1 μM such that the ratio of labeled to unlabeled rA_20_ was around 1:77. Images were taken every minute for 1 h. Upon initial fusion, the droplets do not appear to mix, instead forming spheres but maintaining their segregation (Figure 2A) to the point where, even after over 60 min of fusing, droplets retain visible signatures of multiple previous fusion events (Figure 2B).

To corroborate our observation of slow rA_20_ dynamics, we performed a FRAP assay, analyzing partially bleached droplets with whole droplet radii around 2.0 μm and a bleached ROI radius of around 0.33 μm following 20–30 min of coalescence time. Images were collected every 30 s for 30 min. During this time, very little recovery was observed (Figure 2C), again suggesting limited internal exchange of 6FAM-rA_20_ and overall slow rA_20_ dynamics.

The formation of spherical droplets showing slow scaffold mixing, while less common, is not completely unheard of. Work by Gibson et al. regarding chromatin droplets also showed droplet fusion with slow internal exchange—though notably these droplets did mix by 25 min—which was attributed to the high viscosity and high surface tension of the chromatin condensates formed.^51^ Arrested dynamics of spherical droplets has also been observed in Pab1 droplets which formed spherical droplets and then retained the ringlike structures over 24 h after being sequentially assembled with differently labeled Pab1, albeit with the appearance of reduced fusion over this time as well.^25^ Qualitatively, the polyrA system appears to similarly have very slow exchange of scaffolding monomer in the hour-long window observed, with enough fluidity to minimize surface tension and form spherical droplets along the bottom of the slide. In this way, the polyrA system, like that of the Pab1 droplets, also demonstrates the potential for condensate systems to have time-resolved organization in systems with low mobility in somewhat arrested states.

The observed slow dynamics are interesting not only because they underscore the fact that fusion and spherical droplet formation does not necessarily equate to a liquid-like material state, but also because they support a growing body of research demonstrating the potential for slow RNA dynamics in condensates with strong RNA–RNA networking or due to entanglement effects for long RNA species.^6,15,16,18,29,31,52−55^ Though many of the aforementioned cases deal with networks formed through Watson–Crick base pairs, here we demonstrate that noncanonical RNA condensates are also capable of having somewhat arrested internal dynamics, corroborating previous instances in which long polyA condensates in the presence of Mg^2+^ and PEG demonstrated arrested and incomplete fusion.^16^ While the mechanisms in this case are unclear, previous instances of reduced mobility of long polyrA in condensates has been observed when in the presence of polyArg relative to polyLys which was attributed to increased cation−π interactions in the polyArg case compared to the polyLys case.^16^ While a complex coacervate system is distinct from the rA_20_/Mg^2+^ system here, both instances as well as the arrested fusion observed in long polyrA/Mg^2+^/PEG have polyrA as a slower moving species when compared to other polyrU or polyrC condensates.

### Sodium and Magnesium Cations Interact with Negative Cooperativity on PolyrA Droplet Formation

In previous work focused on the homotypic separation of polyrU in the absence of crowder,^17^ the role of divalent cations was associated with screening backbone charge to enable favorable U–U interactions. To check if Mg^2+^ played a similar role in rA_20_ phase separation, we compared the effect of monovalent salt (Na^+^) on the phase behavior of the rA_20_/Mg^2+^ system. If nonspecific charge screening was the predominant role of the divalent cation, we expected that the addition of significant concentrations of monovalent salt would lower the phase boundary due to the extra ions available to screen backbone charges.

**Figure 3:**
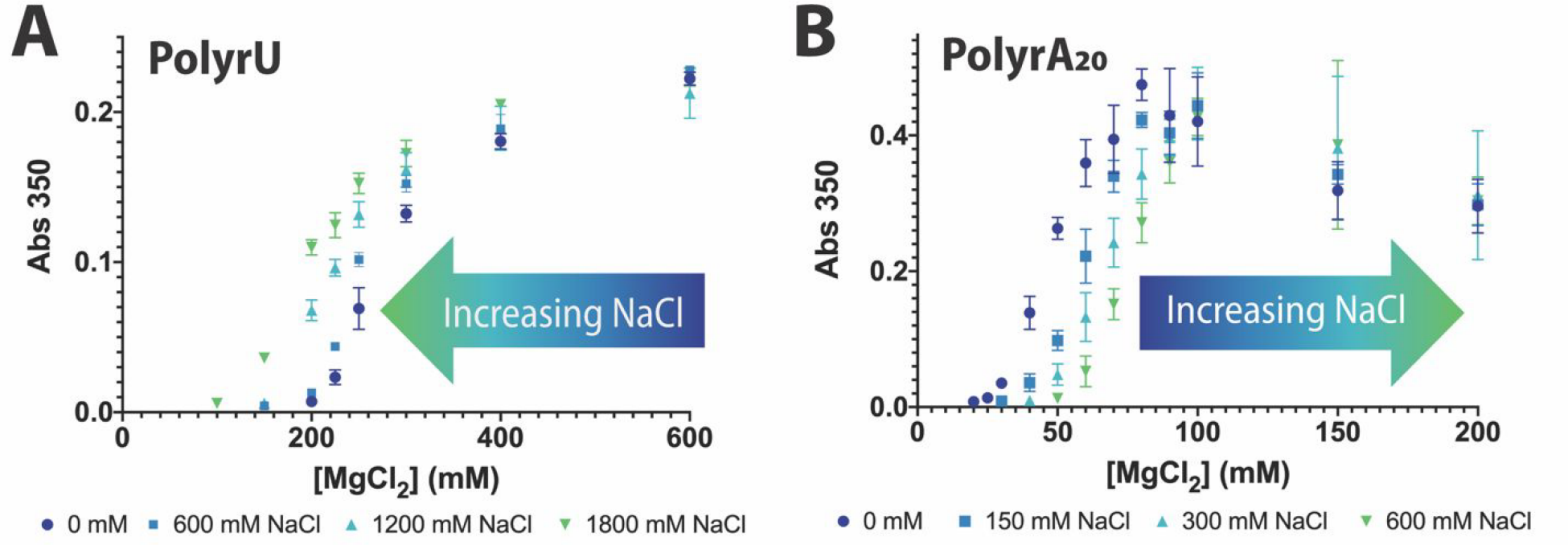
Cooperative effects of Na^+^ and Mg^2+^ for (A) long polyrU and (B) polyrA_20_ condensation (50 mM Tris-HCl, pH 7.5; [RNA] = 0.5 mg/mL). The phase boundary shifts toward higher Mg^2+^ with increasing NaCl for polyrA and to lower Mg^2+^ for polyrU.

Strikingly, when we tested the effects of combinations of Mg^2+^ and monovalent cations, our results revealed opposing cooperativity effects in polyrA condensates compared to those in long polyrU. Where the addition of NaCl was additive in polyrU, shifting phase separation to lower concentrations of MgCl_2_ (Figure 3A), in polyrA systems it was slightly competitive, inhibiting the formation of polyrA condensate formation (Figure 3B). This effect suggests that the role of Mg^2+^ is not merely a general charge screening agent as is hypothesized for polyrU, but that it possibly plays a more specific role that Na^+^ is unable to fulfill and also opposes when competing with Mg^2+^.

Evidence here for opposing cooperativity effects supports polyrA and Mg^2+^ having different interactions compared to other nucleic acid species, which could in turn influence phase separation behaviors. Previous studies have shown that polyrA interacts more directly with Mg^2+^, forming inner sphere contacts compared to polyrU and polydA species which showed reduced dehydration effects consistent with outer sphere and delocalized binding modes, respectively.^41^ The specificity of binding to nucleobases and the distinction between polyrA, polyrU, and polydA fit with the observed distinctions of phase separation properties of polyrA_20_ compared to polyrU_20_ and polydA_20_. The negative cooperativity with Na^+^ is further supported by work which has shown that the polyrA ion atmosphere and structure can change in the presence of Na^+^ compared to Mg^2+^, with Mg^2+^ comparatively correlating with a reduction in A-form helicity of rA_30_ and a decrease in average stack length.^42^ Our work is consistent with the body of literature which suggests that specific Mg^2+^ interactions can influence the properties of polyrA aggregation in a way that is distinct from a purely charge-based contribution.

### PolyrA Can Act as a Phase Separation Promoter

Given the ability of polyrA to undergo phase separating at such low length and concentration thresholds, one question that emerges is the extent to which such an RNA sequence could serve a functionally similar purpose as IDPs or other low complexity domains in promoting or tuning phase separation properties of slightly longer mixed sequences. We expected that if this were true, sections of homopolymer RNA may be able to tune condensates given the properties of their observed pure, short forms. To test this idea, we used a mixed construct containing select oligomer tails (rU_20_, rA_20_, rN_20_) appended to a “generic” 20 nt sequence (N_20_, 5′-CAGCUCCGCAUCCCUUUCCC-3′) of unstructured RNA frequently used in deadenylation studies,^56−61^ to create three constructs: rN_20_ + rN_20_, rN_20_ + rA_20_, and rN_20_ + rU_20_.

**Figure 4:**
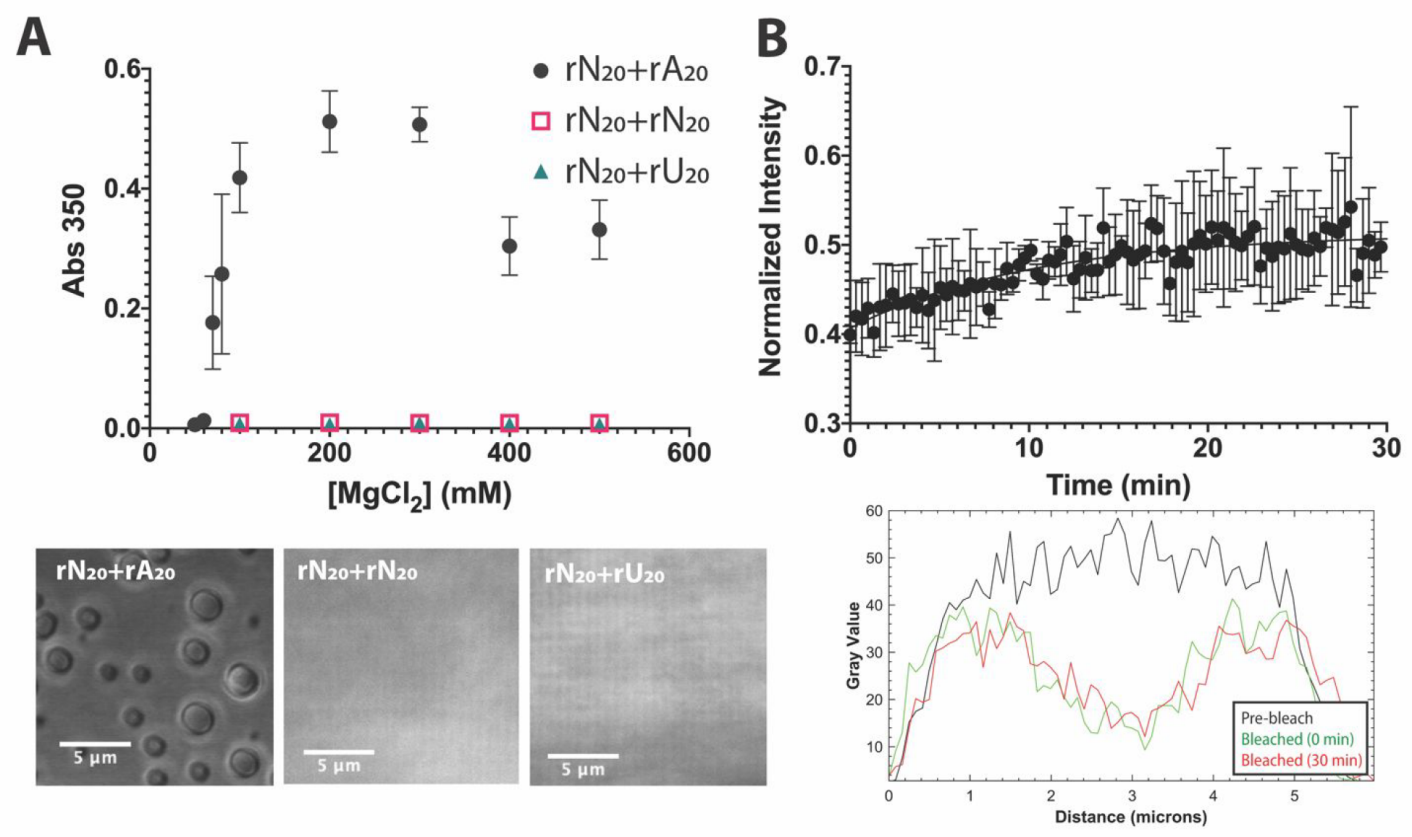
rN_20_ + rA_20_ constructs phase separate and form slowly recovering condensates. (A) Absorption at 350 nm begins to increase around 60 mM MgCl_2_ for rA_20_-tailed constructs ([rN_20_ + rA_20_] = 38.6 μM, 50 mM Tris-HCl, pH 7.5), while similar phase separation is not observed in rN_20_ + rN_20_ or rN_20_ + rU_20_ constructs. (B) Averaged FRAP recovery of partially bleached rN_20_ + rA_20_ droplets shows slow and incomplete recovery at 30 min for a small, circular, bleached region of interest. See Figure S6 for individual traces and fits. Images and FRAP represent observations of condensates formed with 200 mM MgCl_2_.

Our data from turbidity and microscopy show that the polyrA tailed constructs phase separated more readily, while the mixed 40-mer and the polyrU tailed construct did not (Figure 4A). Phase separation for the polyrA tailed construct separated at ≥70 mM MgCl_2_ for an equal number of bases in solution ([40N] = 38.3 μM). This was a slightly higher value relative to the rA_20_ construct alone, which separated around 30 mM ([rA20] = 76.6 μM), but lower than the phase separation propensity of the 0.2 mg/mL rA_20_ (approximately 31 μM) separation, which occurred around 100 mM Mg^2+^. Through the promotion of phase separation enabled by the rA_20_ tail, we show that the phase separation properties of noncanonical, polyrA interactions can promote phase separation of a mixed sequence that otherwise would not have phase separated at the same lengths and concentrations.

FRAP conducted on 6-FAM-rN_20_ + rA_20_ constructs demonstrated very slow recovery over the course of 30 min (Figure 4B). Compared to the rA_20_ FRAP alone, condensates of the 40-mer construct recovered more rapidly, with a mobile fraction of approximately 0.2 ± 0.1 after 30 min based on an exponential fit. While the recovery is notably still slow and occurring over a long time scale with a large immobile fraction observed, RNA in these condensates are slightly more mobile. Thus, it appears as though the addition of the “generic” RNA may increase the fluidity of the droplets due to the addition of RNA that does not interact as strongly as the polyrA homopolymers. In this sense, it is likely that, while polyrA tunes the phase separation propensities of droplets, overall composition rather than only polyrA modulates droplet properties.

## Conclusion

In this work, we note several interesting observations of polyrA phase separation in the presence of Mg^2+^. First, we find that short polyrA phase separates with the addition of Mg^2+^ despite its comparatively short length scale relative to previous reports of homotypic RNA phase separation. The role of Mg^2+^ is somewhat more specific in polyrA than it was in long polyrU droplets, acting with negative cooperativity with Na^+^. Furthermore, we note that, while the system forms spherical condensates, internal exchange and complete mixing occur on a much slower time scale than our experimental time scale. These results demonstrate the potential for incomplete mixing despite a simple composition containing a single macromolecule due to arrested internal monomers and for slowed dynamics through Mg^2+^-mediated, noncanonical RNA–RNA interactions. This highlights the potential for an additional mechanism of spatial organization due to time-resolved phase separation, despite other putative “fluid-like” characteristics such as spherical droplet formation. Finally, we demonstrate that the addition of polyrA “tails” to short homopolymers confers similar phase separation properties, promoting phase separation compared to equivalent length controls at the concentration tested and showing slow, internal dynamics.

The results of this work raise many interesting questions. For one, further research into the mechanism of Mg^2+^-mediated, polyrA separation is needed both with respect to the role of the chemical structure of polyrA and the potential role of Mg^2+^. With respect to the former, it would be interesting, given the chemical similarities, to observe whether polyG has a similar ability to form dropletlike condensates or whether strong aggregation tendencies lead to amorphous and irregular forms. PolyG is well-known for its capacity to form G-quadruplex structures in the presence of a variety of salts, including K^+^, Na^+^, and Sr^2+^.^40,62,63^ Given its ability to self-interact, it is possible that, like polyrA, a combination of those self-interactions and destabilizing effects of Mg^2+^ at high concentrations could also induce a liquidlike state given the right concentrations. It is also possible that the self-interactions could be too strong and subsequently form amorphous and irregular aggregates. Exploring the potential for polyG separation could provide further understanding of how the chemical structures of different nucleobases tune phase properties of RNA.

With respect to the role of Mg^2+^ and other potential mechanistic inquires, further experimental evidence directly probing the mechanism and roles of Mg^2+^ and rA_20_ in enabling phase separation are needed. Based on this work and prior research, there are several potential factors which could be responsible. One potential hypothesis is that Mg^2+^ plays some sort of structural role, altering polyrA conformation. Competitive effects, such as the one observed between Na^+^ and Mg^2+^ in rA_20_ separation, are sometimes related to structure-specific interactions, and divalent cations, particularly Mg^2+^, have long been associated with RNA folding and specific catalysis.^64−66^ Given polyrA’s potential to form structures and interact with itself, it is possible here that Mg^2+^ either facilitates helix formation or interactions of helices or alternatively causes the intramolecular structure to break down and facilitate intermolecular interactions. The breaking of structure for RNA to form extended conformations with arrested RNA components has been observed in computational studies looking at Watson–Crick pairing based RNA–RNA interactions.^29^ Our work could suggest that noncanonical polyrA undergoes a similar extension and structure breaking under sufficiently high concentrations of Mg^2+^, enabling networking via strong intermolecular base stacking that would otherwise, in dilute conditions, have formed secondary structure motifs.

Such a hypothesis, where Mg^2+^ influences structure, would also be consistent with observations by Plumridge et al. where simulations highlighted favoring of Mg^2+^ binding over base stacking for rA_30_.^42^ Alternatively, it could be the case where Mg^2+^ bridges nucleotides or backbones, cross-linking strands in a way that is specific to its identity. Kankia et al. noted that, in polyrA, Mg^2+^ is thought to make inner sphere hydration contacts in polyrA compared to polyrU and polydA.^41^ In this sense, further computational modeling studying the condensation of polyrA in the presence of Mg^2+^ or alternatively further structural work examining changes in the structure of polyrA in solution compared to in condensates could elucidate whether structure formation or structure breaking could play a role in the biophysical mechanism and whether these interactions give rise to some of the arrested dynamics observed here. The above interactions could give rise to extended RNA clusters and networks^5,29,55,67−73^ or entanglement effects^29,55^ also contributing to the observed arrested dynamics.

The effects of heterogeneous interactions could be another area of future exploration. The combined effects of the 40-mer sequences in fluidizing and condensing RNA suggested that overall composition played a role in phase behavior. While previous studies have found and predicted the 20 nt generic sequence to be largely devoid of significant secondary structure,^56^ here, the precise, mechanistic effects of the specific 20 nt sequence on the observed mobility and phase separation propensities of these are unknown. Further efforts to consider the influences of potentially weak or transient RNA interactions, both canonical and noncanonical, as well as whole transcript influences could provide further insight into the various molecular determinants of RNA behavior in condensates.

Finally, the role of polyrA tails in condensates could be an intriguing avenue of research. While our system uses a higher Mg^2+^ concentration than is physiologically common, many cellular granules are still enriched in polyadenylated mRNA transcripts, which—even when not necessarily being the sole driver of phase separation—could potentially influence dynamics and material states. Previous studies of polyrA/polyArg systems had slowed dynamics,^16^ and here we observed very slow dynamics both in droplets containing rA_20_ only and in condensates with a polyrA tail and a mixed sequence. These slow dynamics, especially in the mixed sequence, could have interesting implications for the role of polyrA in possibly tuning the material properties of condensates. Previous work has also observed that polyadenylated mRNA in stress granules has been fairly slow to exchange and diffuse,^74^ though more work would be needed to (a) assess the limits of polyrA as a condensation tag, (b) determine whether these same arrested dynamics occur in more complex and more physiologically relevant systems (for example, lower concentrations of Mg^2+^ and more crowding), and (c) establish whether adenylation or deadenylation significantly alters phase behavior of longer, more complex sequences.

## Supporting Information Available

Description of FRAP imaging and processing; experiments with PEG; multicolor fusion experiments; individual FRAP curves of rN_20_ + rA_20_ (PDF)
Run of the multicolor fusion experiment (AVI)

## Supplementary Material

jp2c05935_si_001.pdf

jp2c05935_si_002.avi
