# Supplementary material for: Short PolyA RNA Homopolymers Undergo Mg2+-Mediated Kinetically Arrested Condensation
Source: J Phys Chem B. 2022 Nov 15;126(46):9715–25. doi: 10.1021/acs.jpcb.2c05935 (PMC9706566; doi:10.1021/acs.jpcb.2c05935)
Supplement: Supplementary file 1 — jp2c05935_si_001.pdf [file jp2c05935_si_001.pdf]

Supporting Information for Publication

Short polyA RNA homopolymers undergo Mg^{2+}-mediated kinetically arrested condensation

Jenna K.A. Tom[#], Paulo L. Onuchic[#], Ashok A. Deniz*

Department of Integrative Structural and Computational Biology, The Scripps Research Institute, 10550 N. Torrey Pines Rd., La Jolla, CA 92037

FRAP Processing

For fluorescence recovery after photobleaching (FRAP) experiments, samples were prepared by mixing a master mix of buffer (100mM Tris-HCl, pH 7.5, Quality Biological), 2x nucleic acid, and 1-2µM FAM-labeled oligomer. 2x$MgCl_2$ was added to the master mix and droplets were formed. The solution was then transferred to 10% Tween-coated, Lab-Tek chambered #1.5 borosilicate coverglass where they were allowed to fuse for 30-40 minutes.

Droplets of radii 3.3-4µm were partially bleached (ROI Area ~ 0.35 um^2) using 4-6 iterative pulses of 100% the 488 and 405 laser power, after which the bleached area was below 50% of its initial signal (typically to 30-40%).

Two reference and two background ROIs of equal size to the bleached ROI were averaged to get reference and background ROI values. Reference and bleached ROI intensities were first background corrected by subtracting corresponding background ROI intensities. The bleached ROI was then normalized relative to pre-bleach values and then referenced relative to the reference ROIs to correct for photobleaching.

$$Normalized\ intensity\ = \frac{I_t^{bleach}}{I_0^{bleach}} \frac{I_0^{ref}}{I_t^{ref}}$$

Equations were fit to an empirical exponential rise curve,

$$I_t\ =\ A(1 - e^{-t/\tau})\ + I_0$$

where I_0 is the offset value due to incomplete bleaching and A is the fit parameter of the curve. For an exponential rise curve, A is approximately equal to the difference between the predicted asymptote value (I_∞) and I_0. As such, the immobile fraction was approximated by IM = A/(1-I_0) which approximates the form, IM = ($I_\infty\ -\ I_0$)/ ($I_i - I_0$), where I_i is the initial pre-bleach value.

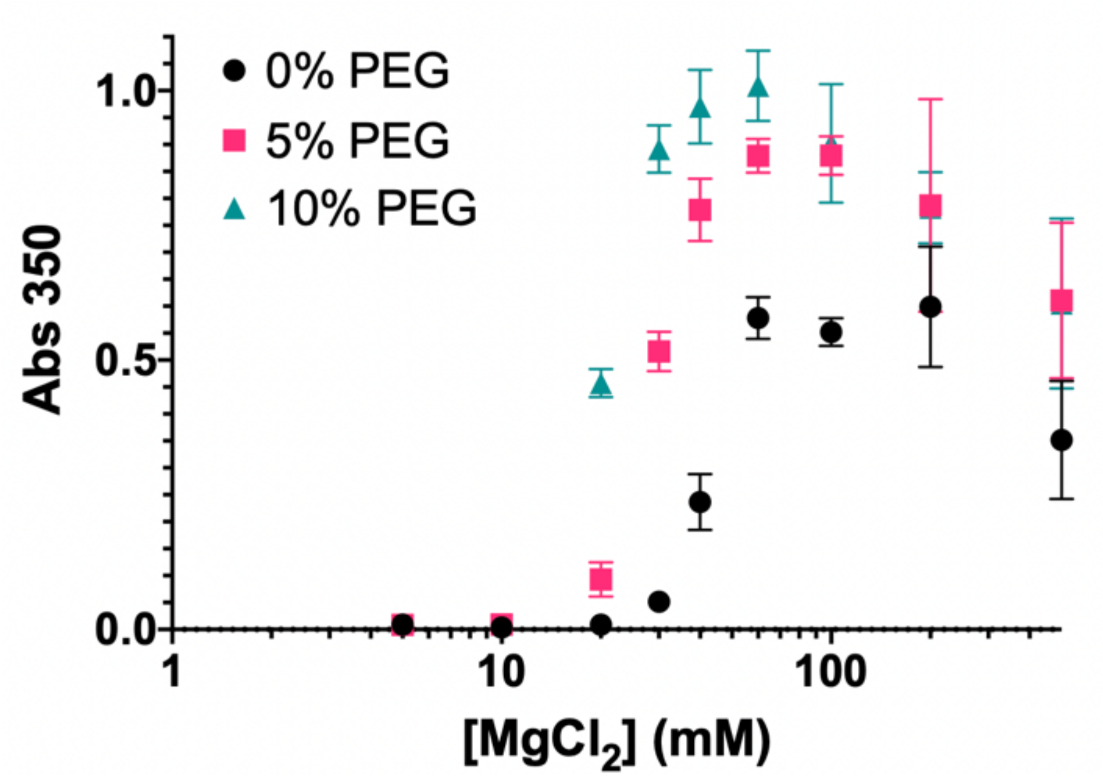

Figure S1. PolyrA20 + PEG. With 5% and 10% PEG, separation happens at 20mM $MgCl_2$

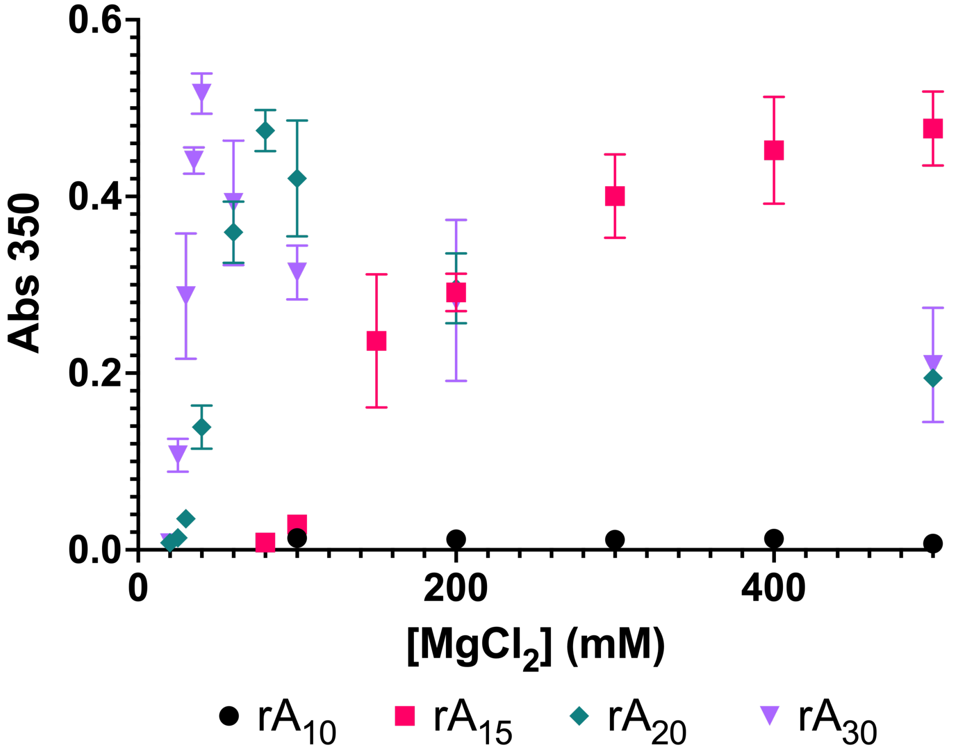

Figure S2. Variations in polyrA length. Threshold Mg^{2+} to induce phase separation decreases with increasing polyrA length. rA_{10} does not undergo phase separation under 500mM Mg^{2+}. Data from this figure is recapitulated in Figure 1D with a truncated x-axis.

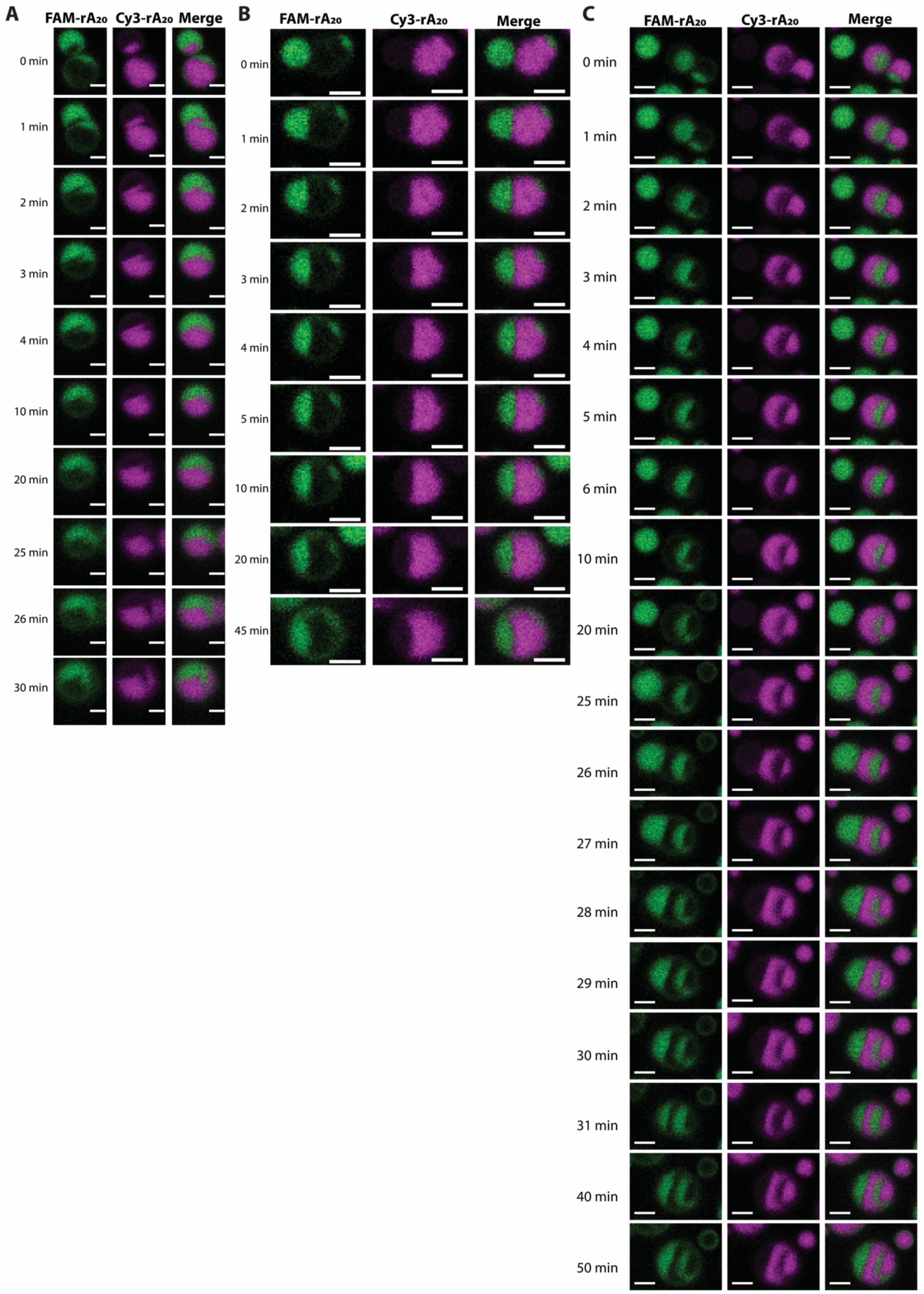

Figure S3. Extended Fusion of droplets. Three examples show the FAM-A20, Cy3-A20, and merge figures at select timepoints (scale bars = 2 μm). Time @ 0 min corresponds to an arbitrary time just before fusion (time post fusion is 22min, 33min, and 34 min for droplets A, B, and C respectively). Panel A is an extended version of what is shown in Fig 2A.

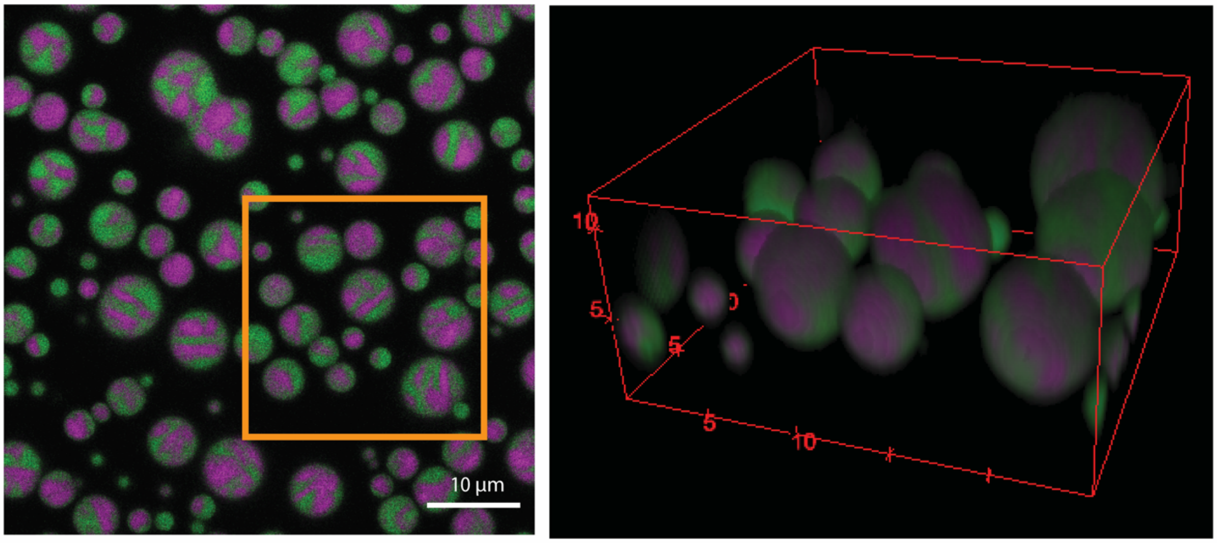

Figure S4. 3D rendering of droplets post-fusion experiment. Confocal z-stack scans were collected at 0.37 µm intervals 70minutes after addition of Cy3-rA_{20} labeled droplets (85 minutes after formation by $MgCl_2$ addition), and rendered (right panel) using the 3D viewer plugin in Fiji. Droplets form spherical shapes despite minimal internal mixing. Left Panel is the same as Fig 2B.

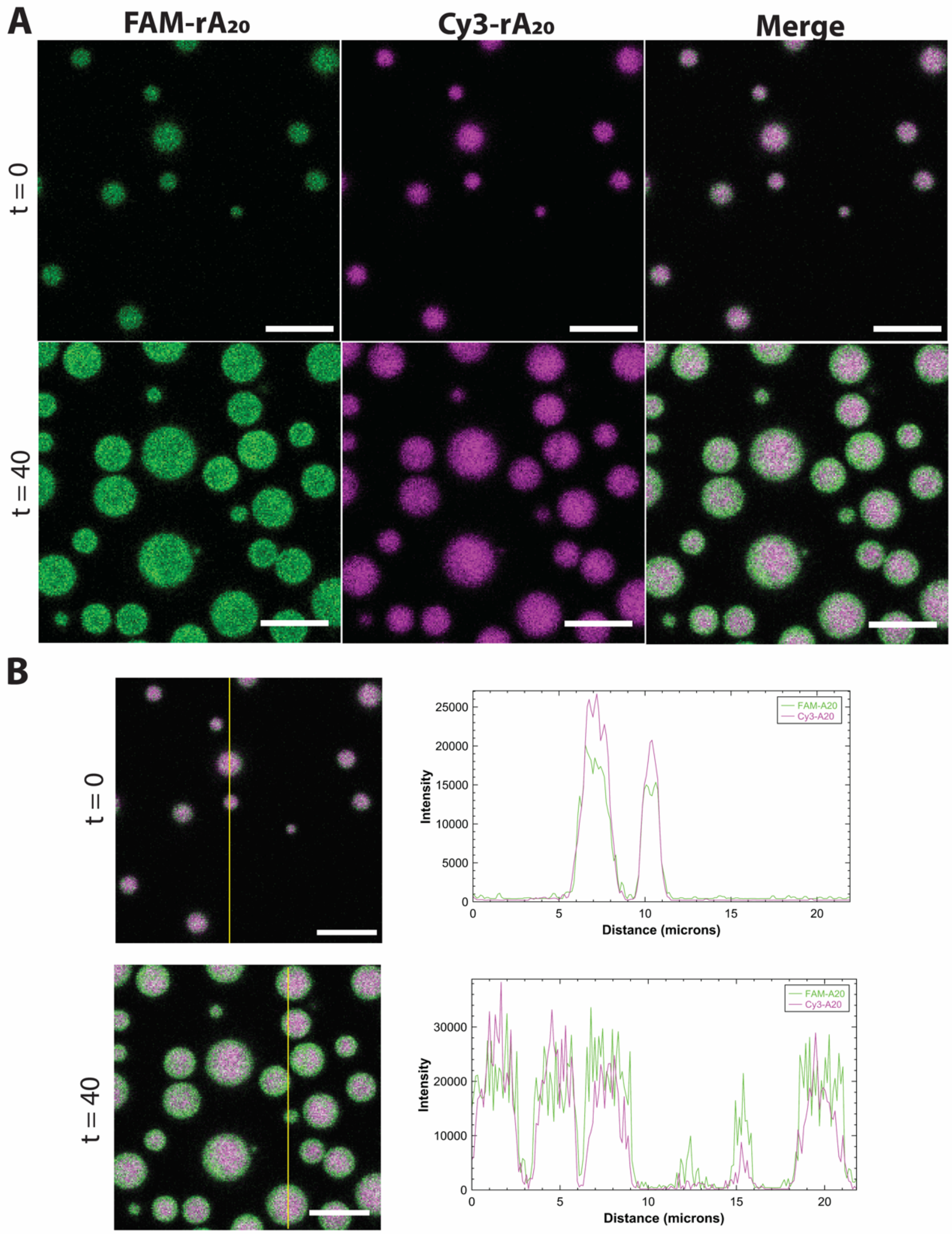

Figure S5. Droplets with both dyes mixed in prior to $MgCl_2$ addition form mixed droplets.

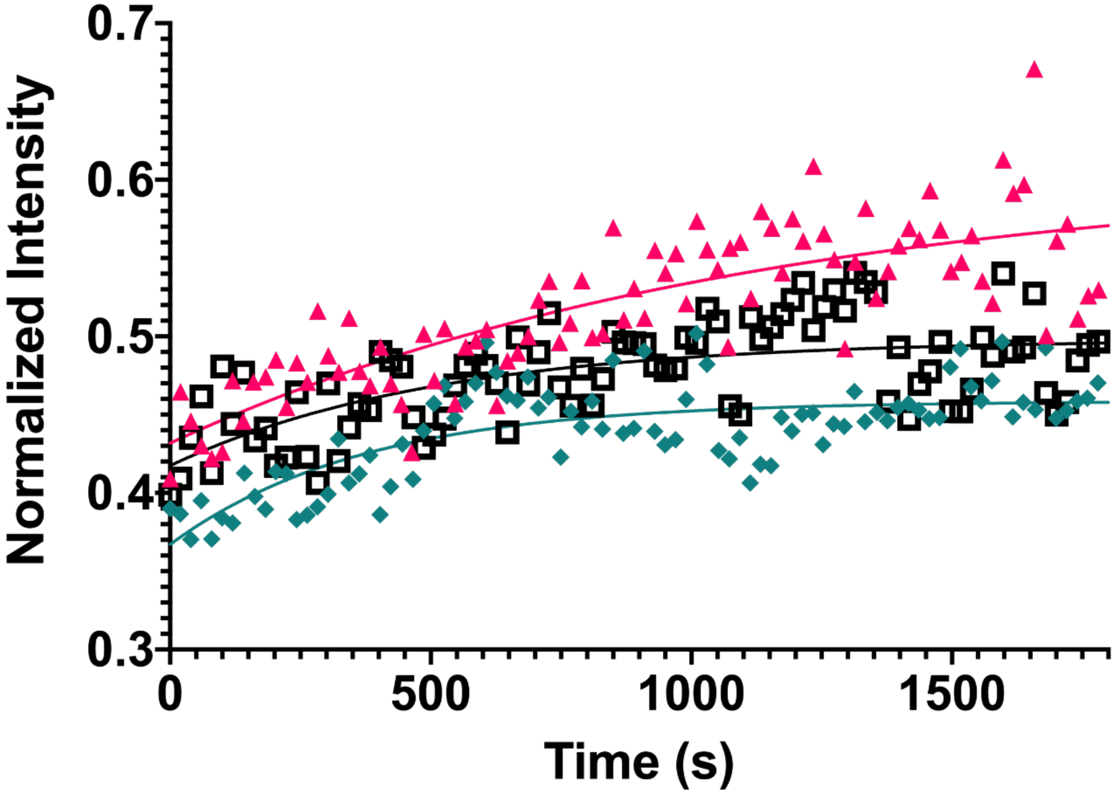

Figure S6. Individual FRAP runs for rN20A20 constructs. Each curve was fit individually to find the plateau value at 30 minutes (1800s).

MOVIE

MOV.S1: Video of a fusion experiment from which Figures 2B, S2B, S2C and S3A originate. Each frame is 1 minute of progression in time. Droplets with Cy3-A20 are added to the top of the droplet by pipet between t = 0 min and t = 1min, which correspond to 14 and 15 minutes after droplet formation respectively. Autofocus using the “Reflection” setting was applied every 5 frames.
